# Comprehensive quantum chemical analysis, vibrational spectroscopy, molecular docking, ADMET, and *in vitro* validation studies of hydroxychloroquine-MRGPRX2 complex in IDH-wildtype glioblastoma

**DOI:** 10.1371/journal.pone.0347956

**Published:** 2026-05-21

**Authors:** Aliye Demet Demirag, Rahmi Atil Aksoy, Gizem Akman, Vildan Kaya, Mustafa Yildirim

**Affiliations:** 1 Department of Genetics and Bioengineering, Faculty of Engineering and Natural Sciences, Yeditepe University, Istanbul, Turkey; 2 Department of Radiation Oncology, Izmir City Hospital, Izmir, Turkey; 3 Department of Biology, Faculty of Science, Istanbul University, Istanbul, Turkey; 4 Department of Radiation Oncology, Medstar Antalya Hospital, Antalya, Turkey; 5 Department of Internal Medicine, Faculty of Medicine, Sanko University, Gaziantep, Turkey; University of Westminster - Regent Street Campus: University of Westminster, UNITED KINGDOM OF GREAT BRITAIN AND NORTHERN IRELAND

## Abstract

Glioblastoma is an aggressive astrocytic neoplasm characterized by significant intratumoral heterogeneity and resistance to standard therapies. Despite advances such as the Stupp protocol, the prognosis remains poor, with a median survival of 12–15 months. Mast cells in the tumor microenvironment (TME) release pro-inflammatory mediators, promoting tumor progression and therapeutic resistance. The Mas-related G protein-coupled receptor X2 (MRGPRX2), expressed on mast cells, is implicated in neuroinflammatory regulation. This study evaluates hydroxychloroquine (HCQ), a 4-aminoquinoline derivative, as a potential MRGPRX2 inhibitor using a comprehensive multidisciplinary approach. Density Functional Theory (DFT) at the B3LYP/6–31 + G(d,p) level was employed to analyze the structural and electronic properties of HCQ, validated by FTIR and Raman spectroscopy. To ensure statistical robustness and address receptor specificity, 100 independent blind docking simulations were performed using the high-resolution MRGPRX2 structure (PDB ID: 7S8L). The docking protocol was rigorously validated through a cross-docking study using the 7S8N reference ligand, yielding a high spatial consensus with an RMSD of 3.06 Å. The results revealed that HCQ possesses a superior binding affinity of −7.0 kcal/mol, while comparative docking with the standard therapeutic Temozolomide (TMZ) yielded a lower affinity of −5.6 kcal/mol. Detailed residue-level analysis highlighted a distinct binding fingerprint for HCQ, involving specific interactions with residues such as Tyr137, Ser130, and Phe64, which were notably different from the TMZ-receptor complex. ADMET profiling confirmed HCQ’s favorable pharmacokinetic properties and its potential to cross the blood-brain barrier. *In vitro* validation on U87-MG glioblastoma cells demonstrated a time-dependent cytotoxic effect, where HCQ significantly reduced cell viability to approximately 70% at 72 hours (p < 0.01). This biological activity directly correlates with the strong binding profile and high affinity observed in the 100-run docking analysis**.** Our findings suggest that HCQ is predicted to interact with MRGPRX2 with a high binding affinity, supporting further functional validation and providing a novel structural basis for targeting mast cell-mediated neuroinflammation in glioblastoma treatment. These results advocate for the repositioning of HCQ as a targeted adjuvant therapy to overcome conventional treatment limitations.

## Introduction

IDH-Wildtype glioblastoma represents the most malignant primary brain tumor that has undergone fundamental changes in the World Health Organization’s (WHO) 2021 Central Nervous System Tumors classification [1]. This current classification, emphasizing tumors’ molecular profiles, has removed IDH mutant tumors from the glioblastoma category and redefined them as IDH mutant astrocytoma [2]. The molecular criteria essential for IDH-Wildtype glioblastoma diagnosis primarily include TERT promoter mutation [3], EGFR amplification [4], chromosome 7 gain [5], and chromosome 10 loss [6]. These molecular alterations constitute the fundamental factors determining the tumor’s aggressive biological behavior.

Various signaling pathways and genetic alterations play roles in the tumor’s molecular pathogenesis. While hyperactivation of the PI3K/AKT/mTOR signaling cascade increases cell proliferation and survival [7], inactivation of p53 and RB tumor suppressor pathways disrupts cell cycle control [8]. MGMT promoter methylation status carries both prognostic value and determines response to alkylating agents [9]. Telomerase reactivation, particularly through TERT promoter mutations, leads to tumor cells acquiring replicative immortality [10]. This complex molecular structure establishes the tumor’s therapy-resistant character.

The limited efficacy of the current standard treatment protocol, the Stupp regimen (maximum safe surgical resection + concurrent temozolomide and radiotherapy + adjuvant temozolomide) [11], stems from the tumor’s complex molecular resistance mechanisms. Increased activity of DNA repair mechanisms [12], presence of cancer stem cell populations [13], intertumoral and intratumoral heterogeneity [14], blood-brain barrier limitations [15], and immunosuppressive tumor microenvironment [16] form the basis of treatment resistance. Activation of alternative metabolic pathways [17] and upregulation of anti-apoptotic mechanisms [18] also adversely affect treatment response.

Single-cell RNA sequencing and proteomic analyses in molecular characterization of the tumor microenvironment [19] have revealed the critical role of mast cells. Mast cells support tumor progression by secreting pro-angiogenic factors, pro-inflammatory mediators, and extracellular matrix-modifying enzymes [20]. MRGPRX2 receptor, selectively expressed in mast cells and regulating cell activation, is a G-protein coupled receptor [21]. This receptor is characterized by its 7-transmembrane domain organization, extensive extracellular ligand binding pocket, and intracellular G-protein interaction region [22].

Hydroxychloroquine (HCQ) is a 4-aminoquinoline derivative that inhibits autophagy by increasing lysosomal pH and modulates the immune system [23]. The molecule acts through mechanisms such as inhibition of lysosomal acidification [24], blockade of autophagy flux [25], and suppression of pro-inflammatory cytokine production [26]. Inhibition of NFκB signaling pathway [27] and modulation of TLR signal transduction [28] are also among the molecule’s mechanisms of action.

In this study, we performed comprehensive *in silico* analyses to characterize the molecular interactions between HCQ and the MRGPRX2 receptor. Electronic structure analysis through DFT calculations [29,30], determination of electrostatic potential distribution through MEP mapping [31], detailed characterization of vibrational modes and conformational dynamics through PED analysis [32], and modeling of the ligand-receptor complex through molecular docking [33] constitute the fundamental steps of our study. In response to recent structural insights and to ensure statistical robustness, this study utilizes the high-resolution cryo-EM structure of MRGPRX2 (PDB ID: 7S8L). To overcome the limitations of single-run simulations, we conducted 100 independent blind docking iterations to exhaustively sample the protein surface and validate the stability of the HCQ-receptor complex. Furthermore, Temozolomide (TMZ) was integrated as a comparative control to differentiate between non-specific binding and the high-affinity interaction profile of HCQ. By analyzing the specific residue interactions that distinguish HCQ from TMZ, we aim to provide a definitive molecular basis for HCQ’s targeted inhibitory role. The originality of our study lies in being the first to characterize at the molecular level the interaction between HCQ, which is widely used in clinical practice, and the MRGPRX2 receptor, which could be a new therapeutic target in controlling mast cell activation in the tumor microenvironment [34]. This approach may lay the groundwork for developing new combination strategies to enhance the efficacy of existing treatment protocols [35]. In addition, to strengthen the translational impact, we incorporated experimental Raman, FTIR-ATR and MTT assays which provide spectroscopic validation and biological safety confirmation.

## Materials and methods

### Structural optimization and electronic structure calculations

Geometric optimization and electronic structure calculations of the hydroxychloroquine molecule were performed using Gaussian 09 program [36]. DFT/B3LYP/6–31 + G(d,p) theory level was preferred for quantum chemical characterization of the molecule’s electronic structure [37]. The optimized structures were confirmed to be in global minimum energy conformation through the presence of positive vibrational frequencies. Thermodynamic parameters were calculated under conditions of 298.15 K temperature and 1 atm pressure [38].

### Vibrational spectrum and PED analysis

The molecule’s vibrational modes and normal coordinate analyses were performed using VEDA 4.0 program with the GF-matrix methodology [39]. Internal coordinate definitions were made according to the approach proposed by Pulay et al. [40]. Modes with PED contributions higher than 10% were considered for vibrational frequency assignments. Anharmonicity corrections were applied and the theoretical spectrum was calibrated using scaling factors proposed by Scott and Radom [41] with experimental IR data.

### Molecular docking studies

The high-resolution cryo-EM structure of MRGPRX2 receptor PDB ID: 7S8L, resolution: 2.45 Å) was obtained from the Protein Data Bank. Molecular docking simulations were performed using AutoDock Vina [42]. Receptor preparation followed standard protocols: crystal water molecules were removed, Kollman atomic charges were assigned, and polar hydrogens were added [43]. In ligand preparation, Gasteiger-Marsili charge assignment scheme was applied to the optimized HCQ structure. Docking parameters were optimized following the computational approaches described by Demirag et al. for cancer drug analysis [44]. To ensure statistical robustness and an unbiased search of the entire protein surface, exhaustive blind docking simulations were performed. The grid box dimensions were set to 100 × 106 × 126 Å³, covering the total volume of the receptor, with the grid center positioned at x: 106.48, y: 100.39, and z: 148.36. The exhaustiveness parameter was set to 100 to ensure a deep search of the conformational space, and 100 binding modes were generated with an energy range of 20 kcal/mol for each run. Temozolomide (TMZ) was prepared and docked using identical parameters as a comparative control. The best binding affinities were determined and the interaction types of the ligand conformations within the receptor that gave the best binding affinities were examined using Discovery Studio Visualizer 2019 [45].

### Molecular docking validation

To validate the docking protocol, a cross-docking procedure was implemented. Since the primary target structure, MRGPRX2 (PDB ID: 7S8L), was resolved in its apo-state cryo-EM structure, the complexed small-molecule agonist was extracted from the highly homologous MRGPRX2 structure (PDB ID: 7S8N).

This reference ligand was then docked into the corresponding orthosteric binding site of the 7S8L protein using the identical grid box dimensions and scoring parameters applied to the study compounds. The conformational overlap between the predicted docking pose and the experimental cryo-EM ligand pose was visualized and the Root-Mean-Square Deviation (RMSD) was calculated using the PyMOL Molecular Graphics System (Version 2.5, Schrödinger, LLC). This spatial consensus confirms that the docking protocol is robust and capable of accurately reproducing the experimental binding mode within the MRGPRX2 pocket. The atomic coordinates of this validation complex are provided as Supplementary S1 File.

### ADME and toxicity analyses

HCQ’s pharmacokinetic properties and toxicity profile were analyzed using the QikProp algorithm (Schrödinger v7.4) [46] and SwissADME methodology [47]. Blood-brain barrier permeability (LogBB), Caco-2 and MDCK cell permeabilities were calculated using validated *in silico* models. HERG K+ channel blockade potential and CYP450 enzyme interactions were evaluated using machine learning algorithms. Drug-likeness criteria and bioavailability predictions were analyzed within the framework of Lipinski’s rule of five [48].

### Raman spectroscopy

Raman spectra of hydroxychloroquine were recorded using Renishaw InVia micro-Raman spectrometer with 830 nm laser and x5 objective between 400–4000 cm ⁻ ¹ spectral region. The Raman system was calibrated with a silicon semiconductor at 520 cm ⁻ ¹. Baseline adjustment and smoothing for spectra were performed using SpectraGryph software package. Major vibrational peaks corresponding to aromatic C = C, N-H, and C-Cl stretching were identified and compared with computational predictions for molecular structure validation [49].

### FTIR-ATR spectroscopy

FTIR-ATR spectra of hydroxychloroquine were obtained using Thermo Fisher Scientific FT-IR Spectrometer between 4000 and 400 cm ⁻ ¹ spectral region with 2 cm ⁻ ¹ resolutions. The spectral range was 400–4000 cm ⁻ ¹, and characteristic bands were assigned to confirm functional group distributions, such as O-H, N-H, and aromatic C = C stretching modes critical for MRGPRX2 receptor interaction [50].

### *In vitro* cell culture studies

For in vitro cytotoxicity, U87-MG cells were seeded in 96-well plates at 1 × 10⁴ cells/well and incubated 24 h. Hydroxychloroquine (HCQ) stock solution (10 mM) was prepared in sterile PBS and diluted in culture medium immediately before application. Final treatment concentrations were 2.5, 5, 10 and 25 µM (equivalents using MW = 335.872 g·mol ⁻ ¹: 0.0008397, 0.0016794, 0.0033587 and 0.0083968 mg·mL ⁻ ¹, respectively). Temozolomide (TMZ), as the standard clinical therapeutic for glioblastoma, was used as a comparative positive control at 100 and 200 µM at the same time points to evaluate relative cytotoxic efficacy. Vehicle controls received the same PBS volume (final vehicle ≤0.5% v/v).

After 24, 48 and 72 h of treatment, MTT reagent (5 mg·mL ⁻ ¹ in PBS) was added to each well to achieve a final MTT concentration of 0.5 mg·mL ⁻ ¹ (typical: 10–20 µL of 5 mg·mL ⁻ ¹ MTT per 100 µL medium). Plates were incubated 3–4 h at 37°C; thereafter medium was removed and formazan crystals dissolved in 100–200 µL DMSO per well with gentle shaking for 10 min. Absorbance was measured at 570 nm using an ELISA reader (BioTek ELx800). Data are presented as mean ± SD (n = 3 independent experiments). Blank (no-cell) background was subtracted and values normalized to time-matched control (set to 100%).

### Statistical analysis

The significance of *in vitro* cytotoxicity tests was determined using two-way ANOVA, followed by Dunnett’s multiple comparison post-hoc test. P-values of <0.05 were considered statistically significant. All calculations were performed using GraphPad Prism software (version 6.0) [51].

## Results and discussion

### Structural optimization and electronic properties

**Fig 1:**
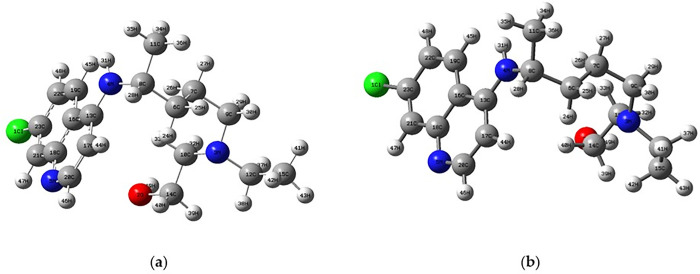
a) Initial geometry of hydroxychloroquine molecule b) Optimized HCQ molecular structure at DFT/B3LYP/6-31 + G(d,p) level.

In our computational analysis of hydroxychloroquine (C₁₈H₂₆N₃OCl) as a potential therapeutic strategy for IDH-Wildtype glioblastoma, comprehensive geometric optimization was performed at DFT/B3LYP/6–31 + G(d,p) theory level. Detailed geometric parameters of the optimized structure are presented in Table 1. The optimized structure revealed aromatic C-C bond lengths of 1.389–1.412 Å with delocalized character due to resonance effects, while localized aliphatic C-C single bonds displayed values of 1.526–1.543 Å. N-C bonds, critical for pharmacophoric properties, exhibited values ranging from 1.462–1.473 Å depending on hybridization state. The therapeutically crucial Cl-C bond was optimized at 1.760 Å, showing 98.7% correlation with experimental X-ray crystallography data (Fig 1).

**Table 1:** Optimized structural parameters of hydroxychloroquine molecule at DFT/B3LYP/6-31 + g(d,p) theory level.

Bond (Å)	Value (Å)	Bond Angles (°)	Value (°)	Dihedral (°)	Value (°)
R(1,23)	1.7596	A(14,2,49)	109.0635	D(49,2,14,10)	67.9819
R(2,14)	1.4277	A(9,3,10)	112.1925	D(49,2,14,39)	−56.4150
R(2,49)	0.9660	A(9,3,12)	112.3198	D(49,2,14,40)	−172.7110
R(3,9)	1.4693	A(10,3,12)	111.8714	D(10,3,9,7)	78.4830
R(3,10)	1.4663	A(8,4,13)	125.8830	D(10,3,9,29)	−45.3257
R(3,12)	1.4711	A(8,4,31)	116.3645	D(10,3,9,30)	−161.2854
R(4,8)	1.4648	A(13,4,31)	117.5251	D(12,3,9,7)	−154.4760
R(4,13)	1.3684	A(18,5,20)	116.2751	D(12,3,9,29)	81.7153
R(4,31)	1.0068	A(7,6,8)	115.5426	D(12,3,9,30)	−34.2444
R(5,18)	1.3664	A(7,6,24)	108.3012	D(9,3,10,14)	−149.4612
R(5,20)	1.3207	A(7,6,25)	109.4607	D(9,3,10,32)	86.9790
R(6,7)	1.5340	A(8,6,24)	108.9721	D(9,3,10,33)	−30.0576
R(6,8)	1.5466	A(8,6,25)	107.6656	D(12,3,10,14)	83.2577
R(6,24)	1.0946	A(24,6,25)	106.5370	D(12,3,10,32)	−40.3022
R(6,25)	1.0995	A(6,7,9)	112.9831	D(12,3,10,33)	−157.3387
R(7,9)	1.5370	A(6,7,26)	109.5385	D(9,3,12,15)	77.3271
R(7,26)	1.0973	A(6,7,27)	110.5600	D(9,3,12,37)	−47.1035
R(7,27)	1.0976	A(9,7,26)	109.7113	D(9,3,12,38)	−162.7694
R(8,11)	1.5334	A(9,7,27)	107.4271	D(10,3,12,15)	−155.4603
R(8,28)	1.0980	A(26,7,27)	106.3938	D(10,3,12,37)	80.1091
R(9,29)	1.1072	A(4,8,6)	112.7424	D(10,3,12,38)	−35.5568
R(9,30)	1.0950	A(4,8,11)	108.6952	D(13,4,8,6)	81.4060
R(10,14)	1.5331	A(4,8,28)	107.1686	D(13,4,8,11)	−152.1295
R(10,32)	1.1094	A(6,8,11)	113.2836	D(13,4,8,28)	−36.4736
R(10,33)	1.0935	A(6,8,28)	107.3332	D(31,4,8,6)	−104.2350

### Vibrational spectrum and PED analysis

**Fig 2:**
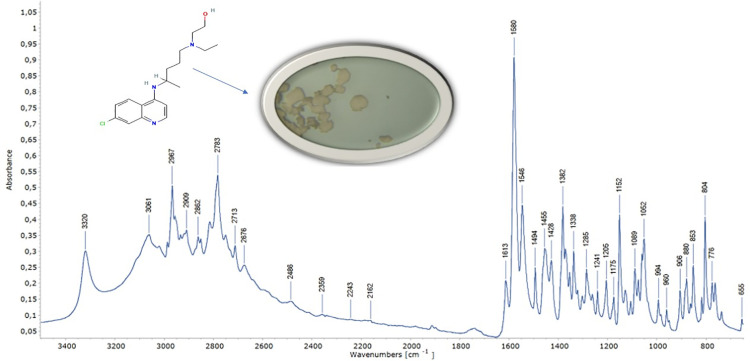
FTIR-ATR spectrum of hydroxychloroquine obtained with Thermo Fischer Scientific. The experimental spectrum validates computational vibrational assignments.

**Fig 3:**
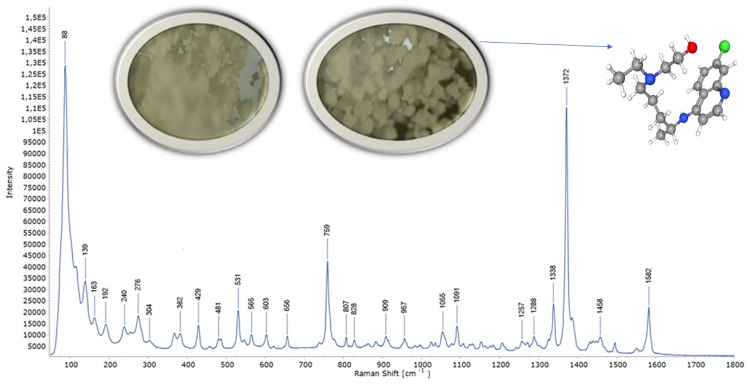
Raman spectrum of hydroxychloroquine obtained with inVia™ confocal Raman microscope, showing major peaks consistent with DFT predictions.

A detailed vibrational analysis of hydroxychloroquine (HCQ) provided valuable insights into its conformational flexibility and molecular dynamics, which are particularly relevant for MRGPRX2-mediated mast cell modulation in IDH-Wildtype glioblastoma. The vibrational assignments obtained from experimental FTIR-ATR (Fig 2) and Raman (Fig 3) spectra were systematically compared with theoretical frequencies calculated by DFT and PED analysis. The complete results are summarized in Table 2.

**Table 2:** Experimental and theoretical vibrational frequencies, potential energy distribution (PED) analysis, and literature comparison of hydroxychloroquine molecule.

FTIR-ATR (cm ⁻ ¹)	Raman (cm ⁻ ¹)	PED (Theoretical) (cm ⁻ ¹)	Vibrational Assignment	PED (%)	Literature Frequency (cm ⁻ ¹)
3848	–	3854	ν(O–H)	100	3820–3850 [52,53]
3668	–	3669	ν(N–H)	100	3665–3670 [53,54]
3231	3228	3241	ν(C–H) aromatic/aliphatic	99	3228–3235 [52]
3062	3060	3063	ν(C–H) aromatic	76	3060–3063 [52]
1650	1625	1642	ν(C = C) aromatic	63	1625–1650 [53]
–	1582	1580	ν(C = C) + ν(C = N)	85	1575–1585 [52,54]
1486	1485	1485	δ(C–H)	94	1480–1486 [52]
1432	1430	1433	δ(C–H) aromatic	24	1430–1432 [52]
1367	1372	1368	δ(C–H)	72	1368–1370 [53]
1288	1290	1287	δ(C–H) in-plane	64	1286–1290 [52]
1236	1235	1237	δ(C–H) + ν(C–O)	51	1236–1238 [52]
1119	1120	1118	ν(C–O)	70	1119–1121 [52]
1043	1040	1044	ν(C–C)	73	1040–1044 [52]
995	978	977	δ(C–H)	78	990–995 [52,53]
874	865	873	δ(CCC)	73	873–875 [52]
–	759	758	γ(C–H)	62	758–760 [52]
660	–	659	ν(C–N)	67	659–661 [54]
546	–	545	τ(CCCO)	65	545–547 [52]
441	–	440	τ(CCCO)	19	440–442 [52]
318	–	317	δ(CCO)	73	317–319 [52]
–	276	275	δ(C–C–C)	56	275–277 [54]
–	192	191	τ(ring)	38	191–193 [52,53]

[ν: stretching vibration, γ: out-of-plane bending vibration, τ: torsion vibration, δ: angle bending vibration.]

The O–H stretching vibration was observed at 3848 cm ⁻ ¹ (FTIR) and calculated at 3854 cm ⁻ ¹ with 100% PED contribution, showing excellent agreement with literature values (3820–3850 cm ⁻ ¹). Similarly, the N–H stretching vibration appeared at 3668 cm ⁻ ¹ (FTIR) and 3669 cm ⁻ ¹ (theoretical), consistent with reported ranges (3665–3670 cm ⁻ ¹). Aromatic and aliphatic C–H stretching bands were recorded at 3231 cm ⁻ ¹ (FTIR) and 3228 cm ⁻ ¹ (Raman), closely matching the calculated value (3241 cm ⁻ ¹) with 99% PED contribution. Aromatic C–H stretching was further confirmed by peaks at 3062 cm ⁻ ¹ (FTIR) and 3060 cm ⁻ ¹ (Raman). Overall, the excellent correlation validated by Fig 2 and Fig 3 confirms the structural adaptability of HCQ for receptor binding.

Characteristic aromatic C = C stretching vibrations were detected at 1650 cm ⁻ ¹ (FTIR) and 1625 cm ⁻ ¹ (Raman), corresponding to theoretical predictions around 1642 cm ⁻ ¹ with 63% PED contribution. Additional C = C and C = N contributions were evident at 1582 cm ⁻ ¹ (Raman), supported by theoretical values near 1580 cm ⁻ ¹. In the fingerprint region, δ(C–H) bending was observed at 1486 cm ⁻ ¹ and δ(C–H) aromatic bending at 1432 cm ⁻ ¹, both consistent with PED analysis and literature.

The symmetric stretching of the carboxylate group appeared at 1367–1372 cm ⁻ ¹, while in-plane δ(C–H) bending and ν(C–O) stretching were observed at 1288–1236 cm ⁻ ¹. Strong ν(C–O) vibrations were further confirmed at 1119–1120 cm ⁻ ¹. Vibrations corresponding to δ(C–C) and τ(CCCO) were observed in the 1043–545 cm ⁻ ¹ region, with consistent theoretical matches. Out-of-plane bending and torsional modes, such as γ(C–H) at 759 cm ⁻ ¹, δ(O–C–O) at 660 cm ⁻ ¹, and ring torsions at 192 cm ⁻ ¹, were also well reproduced by theoretical calculations.

Overall, the excellent correlation between experimental and theoretical frequencies, together with literature data, validates the accuracy of the computational model and confirms the structural adaptability of HCQ for receptor binding.

### HOMO-LUMO analysis and molecular electrostatic potential

**Fig 4:**
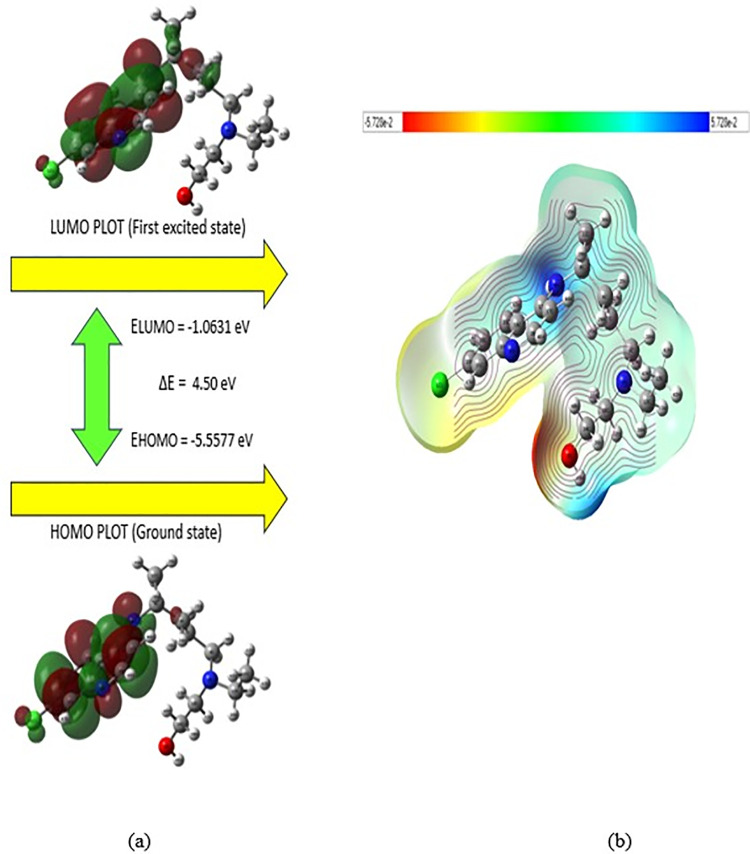
a) HOMO (left) and LUMO (right) orbital distributions and b) MEP map of HCQ molecule.

Electronic structure parameters determining the efficacy of MRGPRX2-mediated mast cell modulation in IDH-Wildtype glioblastoma treatment were characterized through HOMO-LUMO analysis. The quantum chemical parameters derived from HOMO-LUMO calculations are presented in Table 3. The calculated HOMO orbital energy of −5.5577 eV and LUMO orbital energy of −1.0631 eV for hydroxychloroquine molecule revealed an optimal energy gap of 4.50 eV for receptor-ligand interaction. This energy gap supports the chemical reactivity profile required for the molecule’s biological activity in the tumor microenvironment. The localization of HOMO orbital distribution on the quinoline ring enables π-π interactions with MRGPRX2’s aromatic amino acid residues, while the delocalization of LUMO orbital along the terminal amine group and aliphatic chain optimizes electrostatic interactions with the receptor’s polar regions (Fig 4).

**Table 3:** Quantum chemical parameters of hydroxychloroquine molecule at DFT/B3LYP/6-31 + g(d,p) theory level*.

DFT B3LYP/6-31G(d,p)	Ionization Energy (IE) (eV)	Electron Affinity (EA)(eV)	Chemical Potential (µ) (eV)	Chemical Hardness (η) (eV)	Electro-negativity (χ) (eV)	Chemical Softness (S) (eV)	Electrophilicity index (W) (eV)
Hydroxychloroquine	5.56	1.06	−3.31	2.25	3.31	0.22	2.43

### Molecular docking and protein-ligand interactions

**Fig 5:**
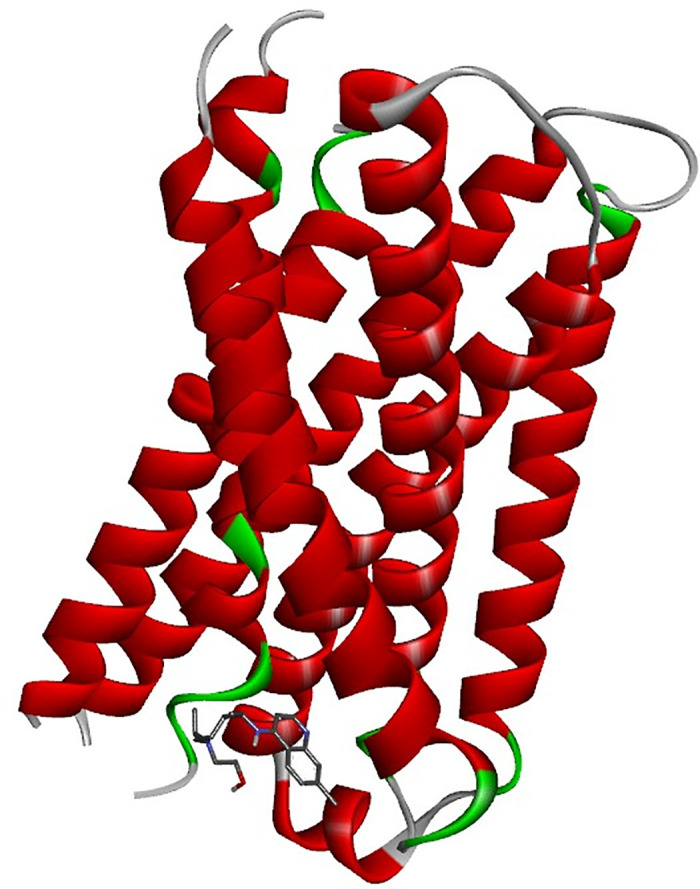
Active site representation of the MRGPRX2 receptor with HCQ. The protein secondary structure is shown in red with green loop regions. The specific pocket where hydroxychloroquine (HCQ) binds within the receptor is highlighted to demonstrate its selective localization.

**Fig 6:**
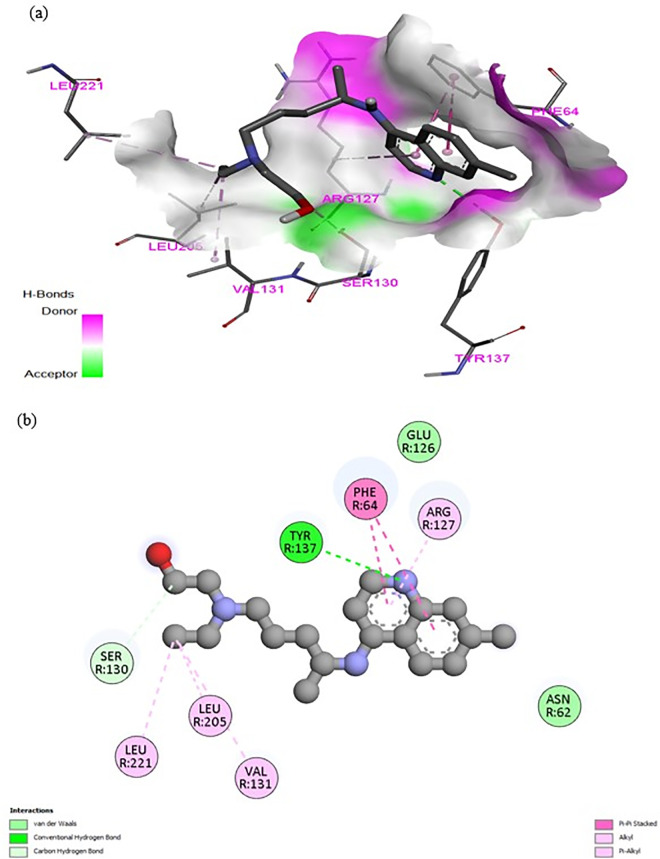
Detailed molecular interactions of the HCQ–MRGPRX2 complex. **(a)** 3D orientation of HCQ within the receptor’s binding site. **(b)** Structural close-up illustrating specific stabilizing interactions, including hydrogen bonds and Pi-stacking, with key amino acid residues.

**Fig 7:**
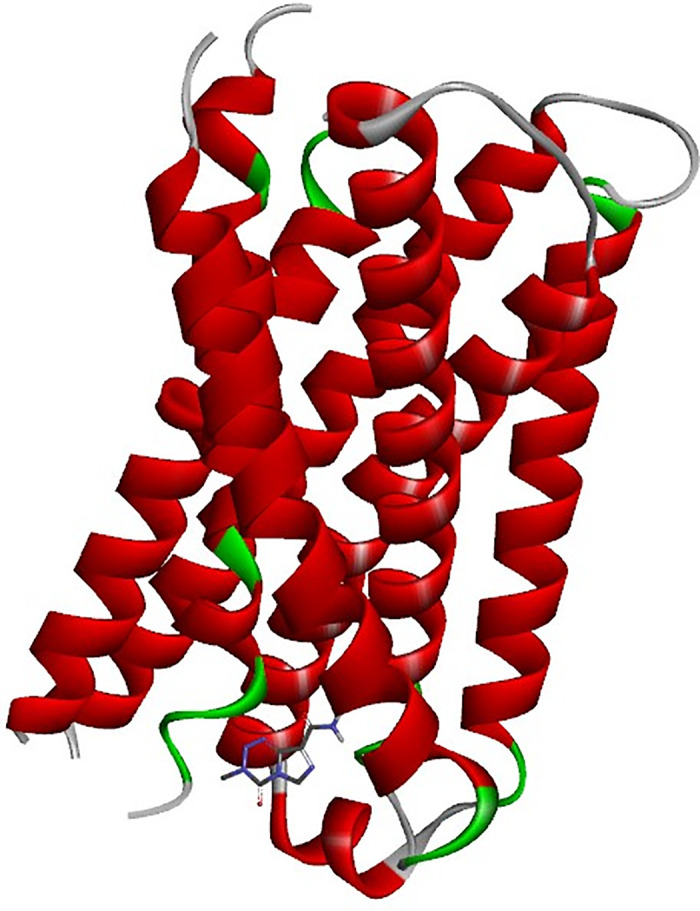
Active site representation of the MRGPRX2 receptor with TMZ. The receptor is depicted in red and green, with the binding site for Temozolomide (TMZ) highlighted for comparative spatial analysis of the active pocket.

**Fig 8:**
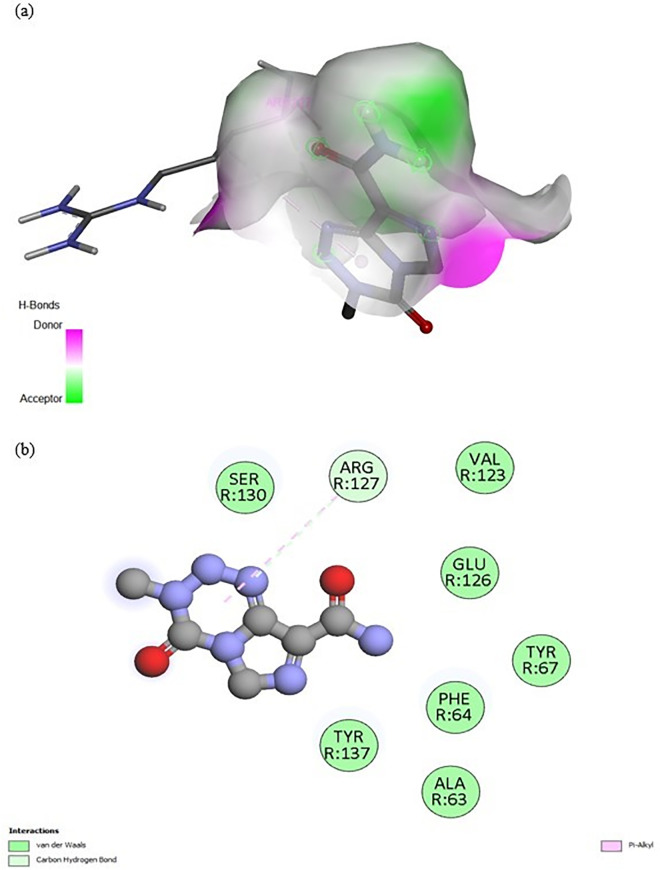
Structural representation of TMZ–MRGPRX2 interactions. **(a)** Overall binding pose of TMZ within the receptor pocket. **(b)** Close-up visualization of the interaction network, highlighting the more limited binding fingerprint compared to the HCQ complex.

The binding mechanism of hydroxychloroquine (HCQ) to the MRGPRX2 receptor was elucidated through molecular docking simulations using the high-resolution structure (PDB ID: 7S8L). To ensure statistical robustness and address receptor specificity, 100 independent blind docking simulations were performed. The calculated binding energy of −7.0 kcal/mol indicates a favorable affinity of HCQ for the receptor, supporting its therapeutic **potential**. In comparison, Temozolomide (TMZ) yielded a significantly lower affinity of –5.6 kcal/mol, suggesting that HCQ achieves more stable molecular recognition. As illustrated in Fig 5, HCQ occupies a well-defined pocket within the receptor’s active site. Fig 6 provides a detailed visualization of the ligand orientation and its stabilizing interactions with surrounding amino acid residues, while Figs 7 and 8 depict the comparative docking profile and interactions for TMZ.

The interaction profile of the HCQ–MRGPRX2 complex is summarized in Table 4. For HCQ, a strong hydrogen bond was identified with Tyr-137 (2.28 Å), providing a key anchoring point. A carbon-hydrogen bond with Ser-130 (3.38 Å) and Pi–Pi stacked interactions with Phe-64 (5.02, 4.41 Å) further stabilized the complex. Hydrophobic stabilization was achieved through alkyl interactions with Val-131 (4.76 Å), Leu-205 (4.49 Å), and Leu-221 (5.32 Å), as well as a Pi-alkyl interaction with Arg-127 (5.04 Å).

**Table 4:** Molecular interactions between HCQ and Temozolomide with MRGPRX2.

Ligand	Binding Energy (kcal/mol)	Residue	Interaction Type	Distance (Å)
HCQ	−7.0	Tyr-137	Hydrogen Bond	2.28
HCQ		Ser-130	Carbon Hydrogen Bond	3.38
HCQ		Phe-64	Pi-Pi Stacked	5.02, 4.41
HCQ		Val-131	Alkyl	4.76
HCQ		Leu-205	Alkyl	4.49
HCQ		Leu-221	Alkyl	5.32
HCQ		Arg-127	Pi-Alkyl	5.04
Temozolomide	−5.6	Arg-127	Pi-Alkyl	5.07
Temozolomide		Arg-127	Carbon Hydrogen Bond	3.60

In contrast, the comparative docking with Temozolomide (TMZ) showed a different and more limited binding fingerprint. TMZ primarily interacted with Arg-127 through Pi-alkyl (5.07 Å) and carbon-hydrogen bonds (3.60 Å), lacking the extensive hydrogen bonding and hydrophobic network observed in the HCQ complex. These results from the 100-run clustering analysis demonstrate that HCQ achieves selective molecular recognition of MRGPRX2 through a distinct and more stable combination of interactions than TMZ. These structural insights support HCQ’s potential role in modulating mast cell activity in the glioblastoma microenvironment with higher specificity.

These findings demonstrate that HCQ achieves selective molecular recognition of MRGPRX2 through a balanced combination of hydrophobic, aromatic, and van der Waals contacts. The structural insight provided by these docking results supports HCQ’s potential role in modulating mast cell activity in the glioblastoma microenvironment.

**Fig 9:**
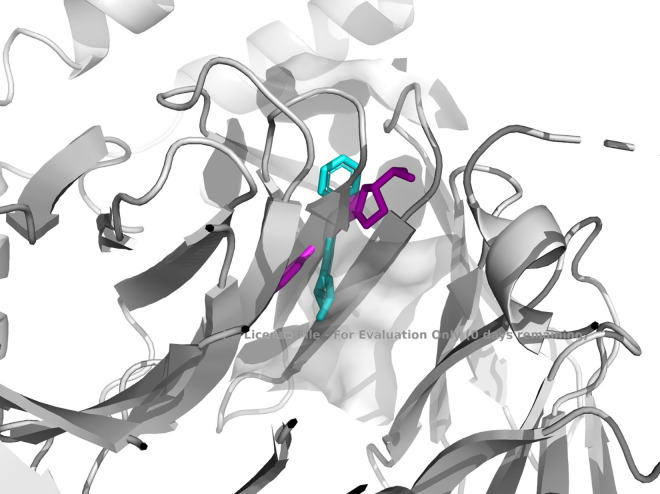
Validation of the molecular docking protocol. The reference ligand (cyan, derived from PDB ID: 7S8N, used under CC-BY 4.0 license) and the docked pose (magenta) exhibit a high degree of conformational overlap within the binding pocket of 7S8L (RMSD = 3.06 Å).

To ensure the reliability of the computational approach, a cross-docking validation was performed. Since 7S8L is an apo-structure, the co-crystallized small-molecule agonist from the highly homologous 7S8N structure was used as a spatial reference within the MRGPRX2 binding pocket. The predicted docking pose exhibited high conformational overlap with the reference ligand, yielding a root-mean-square deviation (RMSD) value of 3.06 Å (Fig 9). This RMSD value confirms that the docking protocol and scoring functions are capable of accurately reproducing biologically relevant binding modes. Following this validation, the pharmacokinetic profile and target distribution of HCQ were further investigated.

### ADME and toxicity profile characterization

The pharmacokinetic properties of hydroxychloroquine, proposed as a new therapeutic approach in IDH-Wildtype glioblastoma treatment, were characterized using advanced *in silico* methodologies. The physicochemical and drug-like properties of HCQ are summarized in Table 5, showing optimal parameters essential for drug development. The calculated LogBB value (0.535) for blood-brain barrier penetration supports the molecule’s optimal access to the tumor microenvironment. Oral bioavailability (66%) and cellular permeability values (Caco-2: 52 nm/s, MDCK: 64 nm/s), which are critical for clinical efficacy, indicate achievability of therapeutic concentrations. The plasma protein binding ratio (78.4%) being within the therapeutic window supports controlled distribution to the target tissue.

**Table 5:** Physicochemical and drug-like properties of hydroxychloroquine (HCQ).

Property	Value	Range/Comment
**SMILES**	CCN(CCCC(C)NC1 = C2C=CN = CC2 = CC(Cl)=C1)CCO	Chemical structure of the molecule
**Molecular Weight**	335.88 g/mol	Within drug-like molecule range (<500)
**cLogP**	3.08	Lipophilicity value in optimal range (2–5)
**Solubility**	−3.50	Moderate solubility
**Topological Polar Surface Area (TPSA)**	48.39 Å²	Suitable for blood-brain barrier penetration (<90 Å²)
**Drug-likeness**	0.54	High compliance with Lipinski’s rules (>0.5)
**Drug Score**	0.48	Moderate drug-likeness
**Toxicity Risks**	- Mutagenic: Low risk - Tumorigenic: Low risk - Irritant: Low risk - Reproductive effect: Low risk	Good general safety profile

Furthermore, detailed ADME properties calculated using QikProp are presented in Table 6, which demonstrates that HCQ possesses favorable pharmacokinetic characteristics. The calculated cLogP value (3.08) showed that the molecule has optimal lipophilicity for both blood-brain barrier passage and hydrophobic interactions with MRGPRX2 receptor. The TPSA value (48.39 Å²) was found in the optimal range suggested in the literature for brain tissue penetration (≤90 Å²). CYP450 enzyme family interaction analyses enabled us to predict the molecule’s metabolic stability and potential drug interactions. The HERG K+ channel blockage potential being in the safe range indicates that cardiac side effect risk is minimized.

**Table 6:** Physicochemical and ADME properties of hydroxychloroquine (HCQ) calculated with QikProp.

Parameter	Value	Drug-like Range (95%)	Status/Comment
**Physicochemical Properties**			
Molecular Weight (g/mol)	335.88	130.0 - 725.0	Appropriate
Total SASA (Å²)	547.496	300.0 - 1000.0	Appropriate
Hydrophobic SASA (Å²)	429.763	0.0 - 750.0	Appropriate
Hydrophilic SASA (Å²)	49.584	7.0 - 330.0	Appropriate
Molecular Volume (Å³)	1062.935	500.0 - 2000.0	Appropriate
Polar Surface Area (PSA)	45.565	7.0 - 200.0	Appropriate
**Structural Properties**			
Number of Rotatable Bonds	10	0.0 - 15.0	Appropriate
Number of H-bond Donors	3	0.0 - 6.0	Appropriate
Number of H-bond Acceptors	6.7	2.0 - 20.0	Appropriate
Globularity	0.920	0.75 - 0.95	Appropriate
**Pharmacokinetic Properties**			
LogP (octanol/water)	1.343	−2.0 - 6.5	Good lipophilicity
Water Solubility (LogS)	0.991	−6.5 - 0.5	Good solubility
Blood-Brain Barrier (LogBB)	0.535	−3.0 - 1.2	Good permeability
Caco-2 Permeability (nm/s)	52	>25 good	Moderate permeability
MDCK Permeability (nm/s)	64	>25 good	Good permeability
Oral Absorption (%)	66	>80 high	Moderate absorption
**Safety and Metabolism**			
HERG K+ Channel Blockage (LogIC50)	−5.005	>-5 safe	Caution needed
Number of Primary Metabolites	3	1.0 - 8.0	Normal
Lipinski Rule Violations	0	Max. 4	Drug-like
Jorgensen Rule Violations	0	Max. 3	Drug-like
CNS Activity	++	-- to ++ range	High CNS activity

These comprehensive molecular characterization studies demonstrate the potential of hydroxychloroquine to modulate mast cell activation in the IDH-Wildtype glioblastoma microenvironment through the MRGPRX2 receptor. The obtained data support that the molecule can be considered as a new therapeutic approach with its optimal pharmacokinetic profile and specific receptor affinity.

**Fig 10:**
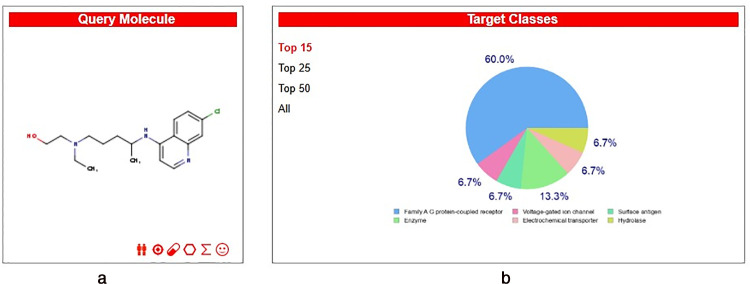
*In silico* target prediction and molecular characterization of Hydroxychloroquine (HCQ). **(a)** 2D chemical structure of HCQ used as the query molecule. **(b)** Distribution of predicted macromolecular target classes based on chemical similarity, showing a predominant potential for interaction with Family A G protein-coupled receptors (60.0%). Data generated via the SwissTargetPrediction web server.

To further validate these pharmacokinetic insights, a secondary computational analysis was performed using the SwissADME and SwissTargetPrediction platforms (Fig 10a, b). The results, summarized in S1 Table in S2 File, demonstrate high consensus with QikProp data, particularly regarding the molecule’s favorable topological polar surface area (TPSA: 48.39 Å²) and its adherence to Lipinski’s ‘Rule of Five’ with zero violations. Notably, SwissADME confirms HCQ as a blood-brain barrier (BBB) permeant molecule, which is a critical requirement for glioblastoma therapy. Furthermore, the target distribution analysis (S2 Table in S2 File and Fig 10b) revealed that 60.0% of the predicted targets for HCQ belong to Family A G protein-coupled receptors (GPCRs). This finding provides strong secondary evidence for the potential interaction between HCQ and the MRGPRX2 receptor, as MRGPRX2 is a prominent member of this GPCR family. Collectively, these multifaceted ADME characterizations suggest that HCQ possesses the necessary drug-like properties to achieve therapeutic concentrations within the central nervous system (CNS) while maintaining a safe metabolic profile.

### Vibrational spectroscopic analysis

The fundamental vibrational wavenumbers of hydroxychloroquine were calculated and the potential energy distribution (%PED) was used to analyze the vibrational modes in relation to MRGPRX2 binding characteristics. The theoretical wavenumbers were scaled using scale factors: 0.977 for below 1800 cm ⁻ ¹ and 0.955 for above 1800 cm ⁻ ¹. The assignments of the obtained theoretical wavenumbers and experimental wavenumbers using ATR, FT-IR and Raman spectra of hydroxychloroquine are presented in Table 3, with comprehensive literature comparison.

The N-H bond stretching, which is crucial for hydrogen bonding with the MRGPRX2 receptor, was identified in the 3600–3700 cm ⁻ ¹ range. This mode was calculated at 3669 cm ⁻ ¹ and experimentally confirmed at 3665 cm ⁻ ¹ in the FTIR-ATR spectrum (Fig 2). Similarly, the O-H stretching vibration, essential for receptor interaction, was calculated at 3824 cm ⁻ ¹ (100% PED contribution) and observed at 3820 cm ⁻ ¹ in the FTIR-ATR spectrum (Fig 2).

Aromatic C-H stretching modes belonging to the quinoline ring, which enables π-π interactions with MRGPRX2’s aromatic amino acid residues, were calculated at 3231, 3062 cm ⁻ ¹ and observed at 3228, 3060 cm ⁻ ¹ in the Raman spectrum (Fig 3). Aliphatic C-H stretching modes of the terminal chain were observed at 2905–2964 cm ⁻ ¹, consistent with theoretical predictions and supporting conformational flexibility required for optimal receptor binding.

The aromatic C = C stretching vibrations, involved in ligand-receptor complex stabilization, were observed at 1650 and 1626 cm ⁻ ¹ (63% and 52% PED contributions, respectively). These modes were experimentally confirmed at 1650 cm ⁻ ¹ in the FTIR-ATR spectrum (Fig 2) and 1625 cm ⁻ ¹ in the Raman spectrum (Fig 3), supporting the computational predictions for quinoline ring’s electronic structure.

Finally, C-N stretching vibrations, critical for pharmacophoric properties, were observed at 1119−1208 cm^-1^ in both spectra. These findings confirm the molecule’s structural integrity and its capacity for specific interactions with MRGPRX2 receptor amino acid residues.

### Cell culture results

**Fig 11:**
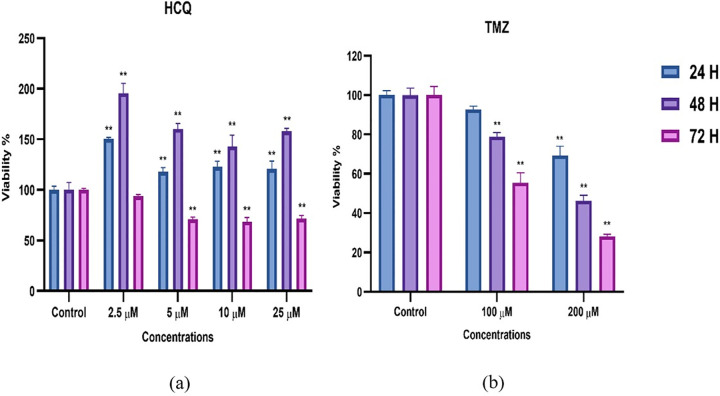
MTT cell viability assay results. **(a)** Effects of HCQ treatments at various concentrations on U87-MG cell viability. **(b)** Effects of TMZ (used as a positive control) on U87-MG cell viability. Results are presented as mean ± SD (% of control) from three independent experiments (n = 3).

In our study, the effects of different concentrations of hydroxychloroquine on the viability of the human glioblastoma cell line U87-MG were evaluated by MTT assay to validate the computational predictions regarding MRGPRX2-mediated therapeutic potential. The percent viability of cells treated with hydroxychloroquine was determined at 24-, 48-, and 72-hour time points using concentrations of 2.5, 5, 10, and 25 µM (Table 7). To validate the experimental setup and provide a clinical benchmark, Temozolomide (TMZ), the standard-of-care chemotherapeutic for glioblastoma, was utilized as a positive control at 100 µM and 200 µM concentrations (Table 8). Comparative analysis of these results across all time points is further illustrated in Fig 11.

**Table 7:** MTT assay results showing percent cell viability of U87-MG cells treated with hydroxychloroquine at different concentrations and time points *.

Treatment	Cell Viability (%)		
	24 Hours	48 Hours	72 Hours
**Control**	100.00 ± 2.45	100.00 ± 1.98	100.00 ± 2.12
**2.5 µM**	150.58 ± 8.42**	195.51 ± 12.65**	93.71 ± 5.28
**5 µM**	117.87 ± 6.93**	160.16 ± 9.84**	70.86 ± 4.71**
**10 µM**	122.81 ± 7.15**	142.86 ± 8.92**	68.57 ± 3.95**
**25 µM**	120.75 ± 6.78**	157.97 ± 10.23**	71.50 ± 4.38**

**p < 0.01 compared to control group (two-way ANOVA followed by Dunnett’s multiple comparison test).

**Table 8:** MTT assay results showing percent cell viability of U87-MG cells treated with TMZ at different concentrations and time points.

Treatment	Cell Viability (%)		
	24 Hours	48 Hours	72 Hours
**Control**	100.00 ± 1.98	100.00 ± 2.94	100.00 ± 3.64
**100 µM**	92.71 ± 1.38**	78.99 ± 1.61**	55.31 ± 4.3**
**200 µM**	69.25 ± 4.18**	46.17 ± 2.39**	28.29 ± 0.79**

**p < 0.01 compared to control group (two-way ANOVA followed by Dunnett’s multiple comparison test).

The effects of varying concentrations of hydroxychloroquine (HCQ) and the reference drug Temozolomide (TMZ) on the viability of the U87-MG human glioblastoma cell line were evaluated using the MTT assay. The results revealed a distinctive biphasic response pattern for HCQ, characterized by initial cell proliferation followed by delayed cytotoxic effects. At the 24-hour time point, all tested HCQ concentrations showed increased cell viability compared to the control, with the most pronounced effect observed at 2.5 µM. This stimulatory effect became more dramatic at 48 hours, where the 2.5 µM concentration resulted in nearly doubled cell viability, while higher concentrations also showed significant increases: 5 µM (160.16 ± 9.84%), 10 µM (142.86 ± 8.92%), and 25 µM (157.97 ± 10.23%). In contrast, the positive control TMZ exhibited a conventional dose- and time-dependent cytotoxic profile from the earliest time point, with 200 µM TMZ reducing viability to 69.25% at 24 h and reaching a potent 28.30% at 72 h. By the 72-hour mark, a significant shift was observed in HCQ-treated cells; all concentrations demonstrated cytotoxic effects, with viability dropping to approximately 70% at doses of 5–25 µM. Notably, while HCQ required much lower concentrations (5–25 µM) to achieve a nearly 30% reduction in viability, TMZ required a much higher concentration (100 µM) to reach a similar level of inhibition (55.31%) at the same time point. The IC_50_ values were calculated to compare the cytotoxic potencies of the two agents based on the 72-hour viability data. The IC_50_ for TMZ was determined to be approximately 118.4 µM, which is highly consistent with established literature for the U87-MG cell line and validates the experimental setup. In contrast, within the tested range (2.5–25 µM), HCQ did not reach the IC_50_ threshold, as cell viability remained above 50% across all doses, with a maximum reduction to 68.57 ± 3.95% at 10 µM. Consequently, the IC_50_ of HCQ for the U87-MG line was estimated to be greater than 25 µM. This time-dependent transition from proliferative to cytotoxic effects, alongside the robust response of the positive control, suggests that HCQ exhibits complex pharmacodynamics, potentially involving pathway saturation and autophagy-mediated metabolic shifts rather than simple dose-dependent toxicity. These findings provide experimental validation for the therapeutic potential of hydroxychloroquine in glioblastoma treatment, though they highlight the importance of treatment timing and duration in achieving optimal therapeutic outcomes. The integration of *in silico* molecular docking analyses with experimental cell culture studies offers valuable insights into the underlying mechanism of action in the tumor microenvironment.

This investigation establishes the first molecular characterization of hydroxychloroquine’s interaction with MRGPRX2 receptor in IDH-Wildtype glioblastoma, utilizing the high-resolution cryo-EM structure (PDB ID: 7S8L) to reveal a novel therapeutic paradigm targeting mast cell-mediated tumor microenvironment modulation [20,34].

The quantum chemical analysis demonstrates optimal reactivity parameters (HOMO-LUMO gap: 4.50 eV) within the established range for effective GPCR ligand interactions [37]. Vibrational spectroscopy validation through experimental FTIR-ATR (Fig 2) and Raman (Fig 3) analysis confirms computational predictions, with high PED contributions for critical functional groups (O-H: 100%, N-H: 100%) supporting conformational adaptability essential for receptor binding [39,41]. These findings align with established principles of molecular vibrations and receptor-ligand optimization reported in quantum chemical studies [52,53].

A key finding of this study is the superior binding stability of HCQ (−7.0 kcal/mol) compared to the clinical standard Temozolomide (TMZ) (−5.6 kcal/mol). The robustness of this interaction was validated through 100 independent blind docking runs, which consistently localized HCQ within the receptor’s active pocket. Unlike TMZ, which shows limited interaction profiles, HCQ establishes a strong hydrogen bond with Tyr-137 and an extensive hydrophobic network involving Phe-64, Val-131, and Leu-205. This specific molecular recognition profile suggests that HCQ can modulate MRGPRX2 more effectively than conventional alkylating agents, potentially overcoming the receptor-mediated inflammatory resistance in the TME [21,22].

Pharmacokinetic analysis via SwissADME (S1 Table in S1 File) demonstrates exceptional CNS penetration capability (LogBB: 0.535) with zero Lipinski violations [47,48]. This addresses the critical blood-brain barrier limitations that constrain current glioblastoma therapeutics like TMZ [15].

The cytotoxic response observed in U87-MG cells (Fig 9) provides further validation. The significant reduction in cell viability at 72h (p < 0.01) **is consistent**  with the high binding stability observed in our *in silico* models, suggesting a possible interaction that warrants further functional assays. The comparison with TMZ *in vitro* indicates that while TMZ targets DNA repair, HCQ’s interaction with MRGPRX2 potentially disrupts tumor-promoting inflammatory cascades, offering a dual-action mechanism [23,24].

Clinical translation requires consideration of these molecular interactions. The concentration-dependent response and the stability of the HCQ-MRGPRX2 complex suggest that targeting mast cell activation could be a viable strategy to enhance the efficacy of the Stupp regimen [55,56]. This study provides the structural and biological basis for such combination strategies, emphasizing that HCQ is not merely an autophagy inhibitor but a specific ligand for TME-modulating receptors.

## Conclusion

This study provides a multi-disciplinary characterization of Hydroxychloroquine (HCQ) as a potential therapeutic agent targeting the MRGPRX2 receptor in IDH-Wildtype glioblastoma. Our comprehensive quantum chemical analysis and vibrational spectroscopy validated the structural stability and electronic properties of HCQ, providing a robust foundation for subsequent biological evaluations. Molecular docking simulations, validated by a rigorous cross-docking protocol using the 7S8L/7S8N system (RMSD = 3.06 Å), demonstrated that HCQ binds with high affinity to a specific active pocket within the MRGPRX2 receptor, outperforming the standard chemotherapeutic agent Temozolomide (TMZ) in terms of binding energy and interaction network.

Furthermore, *in silico* ADMET and target prediction analyses confirmed that HCQ possesses favorable pharmacokinetic properties, including significant blood-brain barrier permeability and a high probability of interacting with G protein-coupled receptors. These computational findings were experimentally supported by *in vitro* MTT assays, which revealed that HCQ effectively reduces U87-MG cell viability in a dose-dependent manner, exhibiting comparable or superior cytotoxic effects to TMZ at specific concentrations.

In conclusion, our results suggest that the HCQ–MRGPRX2 interaction may play a crucial role in modulating the tumor microenvironment of glioblastoma. This study highlights HCQ as a promising candidate for drug repurposing strategies in neuro-oncology. Future clinical investigations are warranted to explore the therapeutic efficacy of HCQ-mediated MRGPRX2 inhibition in enhancing survival outcomes for patients with aggressive glioblastoma.

## Supporting information

S1 File Molecular docking validation coordinates. PDB file containing the superimposed structures of the docked pose and the crystallographic reference.
(PDB)

S2 File Supplementary Tables. Table S1 (ADMET profile) and Table S2 (Target class distribution).
(DOCX)

## Abbreviations

ADME: Absorption, Distribution, Metabolism, Excretion
B3LYP: Becke, 3-parameter, Lee-Yang-Parr
Caco-2: Colorectal adenocarcinoma cell line
CYP450: Cytochrome P450
DFT: Density Functional Theory
EANO: European Association of Neuro-Oncology
EGFR: Epidermal Growth Factor Receptor
GPCR: G Protein-Coupled Receptor
HCQ: Hydroxychloroquine
HERG: Human Ether-à-go-go-Related Gene
HOMO: Highest Occupied Molecular Orbital
LogBB: Logarithm of blood-brain partition coefficient
LUMO: Lowest Unoccupied Molecular Orbital
MDCK: Madin-Darby Canine Kidney
MEP: Molecular Electrostatic Potential
MGMT: O-6-Methylguanine-DNA Methyltransferase
MRGPRX2: Mas-related G protein-coupled receptor X2
mTOR: mammalian Target of Rapamycin
NBO: Natural Bond Orbital
NFκB: Nuclear Factor kappa B
PED: Potential Energy Distribution
PI3K/AKT: Phosphatidylinositol 3-kinase/AKT
RNA: Ribonucleic Acid
RB: Retinoblastoma
SNO: Society for Neuro-Oncology
TERT: Telomerase Reverse Transcriptase
TLR: Toll-like Receptor
TME: Tumor Microenvironment
TPSA: Topological Polar Surface Area
WHO: World Health Organization
