# Supplementary material for: Comprehensive quantum chemical analysis, vibrational spectroscopy, molecular docking, ADMET, and in vitro validation studies of hydroxychloroquine-MRGPRX2 complex in IDH-wildtype glioblastoma
Source: PLoS One. 2026 May 21;21(5):e0347956. doi: 10.1371/journal.pone.0347956 (PMC13193503; doi:10.1371/journal.pone.0347956)
Supplement: S2 File — Table S1 (ADMET profile) and Table S2 (Target class distribution). (DOCX) [file pone.0347956.s002.docx]

**Table S1.** Comprehensive ADMET profile of hydroxychloroquine from SwissADME analysis.

| **Parameter** | **Value** | **Optimal Range/Status** |
| --- | --- | --- |
| **PHYSICOCHEMICAL PROPERTIES** |  |  |
| Molecular Formula | C₁₈H₂₆ClN₃O | - |
| Molecular Weight | 335.87 g/mol | (<500) |
| Heavy Atoms | 23 | - |
| Aromatic Heavy Atoms | 10 | - |
| Fraction Csp³ | 0.50 | (>0.25) |
| Rotatable Bonds | 9 | (<10) |
| H-bond Acceptors | 3 | (<10) |
| H-bond Donors | 2 | (<5) |
| Molar Refractivity | 98.57 | (40-130) |
| TPSA | 48.39 Ų | (<140) |
| **LIPOPHILICITY** |  |  |
| Log P (iLOGP) | 3.58 | - |
| Log P (XLOGP3) | 3.58 | - |
| Log P (WLOGP) | 3.59 | - |
| Log P (MLOGP) | 2.35 | - |
| Log P (SILICOS-IT) | 3.73 | - |
| **Consensus Log P** | **3.37** | **Optimal** |
| **WATER SOLUBILITY** |  |  |
| Log S (ESOL) | -3.91 | Soluble |
| Solubility (ESOL) | 4.17×10⁻² mg/ml | - |
| Log S (Ali) | -4.28 | Moderately soluble |
| Solubility (Ali) | 1.75×10⁻² mg/ml | - |
| Log S (SILICOS-IT) | -6.35 | Poorly soluble |
| **PHARMACOKINETICS** |  |  |
| GI Absorption | High | Excellent |
| BBB Permeant | Yes | CNS penetration |
| P-gp Substrate | No | No efflux |
| CYP1A2 Inhibitor | Yes | DDI risk |
| CYP2C19 Inhibitor | No | - |
| CYP2C9 Inhibitor | No | - |
| CYP2D6 Inhibitor | Yes | DDI risk |
| CYP3A4 Inhibitor | No | - |
| Log Kₚ (Skin Permeation) | -5.81 cm/s | - |
| **DRUG-LIKENESS** |  |  |
| Lipinski Rule | Yes; 0 violation | Drug-like |
| Ghose Rule | Yes |  |
| Veber Rule | Yes |  |
| Egan Rule | Yes |  |
| Muegge Rule | Yes |  |
| Bioavailability Score | 0.55 | 55% |
| **MEDICINAL CHEMISTRY** |  |  |
| PAINS Alert | 0 | Clean |
| Brenk Alert | 0 | Clean |
| Leadlikeness | No; 2 violations | Rotors>7, LogP>3.5 |
| Synthetic Accessibility | 2.82/10 | Easy synthesis |

*****TPSA, topological polar surface area; GI, gastrointestinal; BBB, blood-brain barrier; P-gp, P-glycoprotein; CYP, cytochrome P450; PAINS, pan-assay interference compounds; GBM, glioblastoma multiforme.

**Table S2.** Target class distribution for hydroxychloroquine from SwissTargetPrediction.

| **Target Class** | **Percentage** | **Examples** |
| --- | --- | --- |
| Family A G protein-coupled receptor | 60.0% | MRGPRX2, MRGPRX1, GPCRs |
| Enzyme | 13.3% | Various enzymes |
| Surface antigen | 6.7% | Cell surface proteins |
| Electrochemical transporter | 6.7% | Ion channels |
| Hydrolase | 6.7% | Hydrolytic enzymes |
| Family C G protein-coupled receptor | 6.7% | Metabotropic receptors |

*The predominance of Family A GPCRs (60%) as predicted targets supports the biological relevance of MRGPRX2 as an HCQ target. This receptor family includes MRGPRX2/MRGPR members involved in mast cell regulation and neuroinflammatory responses.
